# L/N-Type Calcium Channel Blocker Cilnidipine Added to Renin-Angiotensin Inhibition Improves Ambulatory Blood Pressure Profile and Suppresses Cardiac Hypertrophy in Hypertension with Chronic Kidney Disease

**DOI:** 10.3390/ijms140816866

**Published:** 2013-08-16

**Authors:** Tomohiko Kanaoka, Kouichi Tamura, Hiromichi Wakui, Masato Ohsawa, Kengo Azushima, Kazushi Uneda, Ryu Kobayashi, Tetsuya Fujikawa, Yuko Tsurumi-Ikeya, Akinobu Maeda, Mai Yanagi, Yoshiyuki Toya, Satoshi Umemura

**Affiliations:** Department of Medical Science and Cardiorenal Medicine, Graduate School of Medicine, Yokohama City University, 3-9 Fukuura, Kanazawa-ku, Yokohama 236-0004, Japan; E-Mails: to_kana8@yokohama-cu.ac.jp (T.K.); hiro1234@yokohama-cu.ac.jp (H.W.); t106008a@yokohama-cu.ac.jp (M.O.); t116002f@yokohama-cu.ac.jp (K.A.); k_uneda@yokohama-cu.ac.jp (K.U.); t136034d@yokohama-cu.ac.jp (R.K.); tftf@yokohama-cu.ac.jp (T.F.); ytsurumi@yokohama-cu.ac.jp (Y.T.-I.); t096054a@yokohama-cu.ac.jp (A.M.); mai_mew963@hotmail.com (M.Y.); ystoya@yokohama-cu.ac.jp (Y.T.); umemuras@med.yokohama-cu.ac.jp (S.U.)

**Keywords:** ambulatory blood pressure, calcium channel blockers, cardiac hypertrophy, chronic kidney disease, heart rate variability, hypertension (kidney)

## Abstract

Ambulatory blood pressure (BP) and heart rate (HR) profile are proposed to be related to renal deterioration and cardiovascular complication in hypertension and chronic kidney disease (CKD). In this study, we examined the beneficial effects cilnidipine, a unique L/N-type calcium channel blocker (CCB), in addition to renin-angiotensin system inhibitors, on ambulatory BP and HR profile, as well as cardiorenal function in hypertensive CKD patients. Forty-five patients were randomly assigned to the cilnidipine replacement group (*n* = 21) or the control CCBs group (*n* = 24) during a 24-week active treatment period. Although clinical BP values were similar in the cilnidipine and control CCBs groups after the treatment period, the results of ambulatory BP monitoring showed that the 24-h and daytime systolic BP levels in the cilnidipine group were significantly lower compared with the control group after the study. Furthermore, the left ventricular mass index (LVMI) was significantly decreased in the cilnidipine group compared to the control group after the study (LVMI, 135.3 ± 26.4 *versus* 181.2 ± 88.4, *p* = 0.031), with a significant difference in the changes in the LVMI between the cilnidipine and control groups (change in LVMI, −12.4 ± 23.7 *versus* 26.2 ± 64.4, *p =* 0.007). These results indicate that cilnidipine is beneficial for the suppression of pathological cardiac remodeling, at least partly, via a superior improving effect on ambulatory BP profile compared with control CCBs in hypertensive CKD patients.

## 1. Introduction

Hypertensive chronic kidney disease (CKD) patients are reportedly increasing in number, and cardiovascular complications are the most common cause of death in these patients. Thus, it would be a considerable advance in the management of this condition to elucidate the mechanisms involved in the renal deterioration and the cardiovascular events associated with hypertension complicated by CKD and to identify therapeutic approaches to treat them. Recent evidence has indicated that ambulatory, as well as clinical blood pressure (BP) is important for a proper estimation of BP control. In particular, ambulatory BP monitoring has allowed an accurate diagnosis of hypertension [1,2] and determination of the BP and heart rate (HR) circadian rhythms under different pathophysiological conditions, including hypertension and CKD, and may afford a more accurate prognosis than the clinical BP alone. [3–5] The circadian BP pattern in hypertensive patients with CKD has been found to exhibit a blunted nocturnal decrease in BP, and this blunting is associated with autonomic neuropathy and nephropathy [6,7].

In addition to the critical role of the activation of the renin-angiotensin system in the pathogenesis of CKD and the ensuing cardiovascular complications, activation of the sympathetic nerve system is suggested to be involved in CKD and the related cardiovascular complications [8–11]. Cilnidipine is one of the calcium channel blockers (CCBs) and is reported to block two different calcium channels, L-type calcium channels in vascular smooth muscle, which exerts an antihypertensive effect similar to the L-type channel blockers, such as amlodipine, and N-type calcium channels in sympathetic nerve endings, which suppresses increases in sympathetic activity in response, such as vasodilation induced by blockade of L-type calcium channels in vascular smooth muscle [12–14]. In this manner, cilnidipine is suggested to be an effective CCB in combination with inhibitors of the renin-angiotensin system as an anti-hypertensive medication used to inhibit CKD progression and cardiovascular complications. In this study, we compared the therapeutic effects of L/N-type CCB cilnidipine with other CCBs on the ambulatory BP and HR profile and on cardiorenal function in hypertensive CKD patients who had already received antihypertensive therapy comprised of renin-angiotensin system inhibitors (angiotensin receptor blockers, ARBs; angiotensin-converting enzyme inhibitors, ACEIs; and/or direct renin inhibitor, DRI) and a CCB other than cilnidipine.

## 2. Results

### 2.1. Patient Characteristics

Forty-five hypertensive CKD patients, who were already receiving antihypertensive treatment comprised of renin-angiotensin system inhibitors (angiotensin receptor blockers, ARBs; angiotensin-converting enzyme inhibitors, ACEIs; and/or direct renin inhibitor, DRI) and one of the CCBs other than cilnidipine at a standard dose for a period of more than four weeks, were enrolled from April, 2008, to November, 2011. The causes of CKD were hypertensive nephrosclerosis (*n* = 29), diabetic nephropathy (*n* = 10), chronic glomerulonephritis (*n* = 4), polycystic kidney disease (*n* = 1) and chronic pyelonephritis (*n* = 1). The patients were randomly assigned to the cilnidipine group (*n* = 21) or the control group (*n* = 24). In the control CCBs group, the CCBs continued during the study period were amlodipine (*n* = 17), nifedipine (*n* = 3), azelnidipine (*n* = 2), benidipine (*n* = 1) and manidipine (*n* = 1). Four patients in the cilnidipine group and two patients in the control group discontinued participation in the study, due to a referral to another hospital. There was no significant difference in the baseline characteristics, including the cause of CKD, and medications, including the renin-angiotensin system inhibitors (ARBs, ACEIs and/or DRI) used during the study between the two groups (Table 1). Cilnidipine replacement and control CCBs therapy were well-tolerated without any significant adverse events, and the average dose of cilnidipine was 14.4 ± 6.1 mg daily after a period of 24 weeks of treatment.

### 2.2. Effects of Cilnidipine on Ambulatory BP Profile

In both groups, the anti-hypertensive therapy was aimed at achieving the clinical BP goal (BP < 130/80 mmHg), but not the ambulatory BP goal, and thus, the results indeed showed that the mean clinical BP values were similar in the cilnidipine and CCBs control groups at baseline and after the 24-week active treatment period (Table 2). With respect to the ambulatory BP profile, 24-h, daytime and nighttime systolic/diastolic BP and HR were comparable in the cilnidipine and control groups at baseline (Table 3). However, compared with the control CCBs group, the 24-h and daytime systolic BP levels in the cilnidipine group were significantly lower after the 24-week treatment period (Table 3). In addition, 24-h systolic BP variability increased in the control group and decreased in the cilnidipine group after the study period, and the difference in these changes was significant (Table 3; change in 24-h systolic BP variability, 1.3 ± 2.9 *versus* −1.3 ± 3.1, *p =* 0.009). Furthermore, there were similar increases in the 24-h, daytime and nighttime HR in the control group, but decreases in the cilnidipine group, and the differences in these changes were also significant (Table 3; change in 24-h HR, 2 ± 5 *versus* −5 ± 9, *p <* 0.001; change in daytime HR, 3 ± 12 *versus* −4 ± 6, *p* = 0.008; change in nighttime HR, 3 ± 6 *versus* −1 ± 5, *p =* 0.034).

### 2.3. Effects of Cilnidipine Substitution on Renal Function, Renin-Angiotensin System, Systemic Sympathetic Nerve Activity, Inflammation and Oxidative Stress

The renal function parameters (serum creatinine, estimated glomerular filtration rate (eGFR), urinary protein/creatinine ratio (UPCR) and urinary type IV collagen) were similar in the cilnidipine and control groups at baseline and after the 24-week active treatment period (Table 4). The endocrine function parameters, including two markers of the renin-angiotensin system (plasma renin activity (PRA) and plasma aldosterone concentration (PAC)) and a very weak index of systemic sympathetic nerve activity (noradrenaline) were also similar in the cilnidipine and control groups at baseline and after the study period (Table 4). Furthermore, markers of inflammation and oxidative stress (high sensitive C-reactive protein (hs-CRP), pentosidine, MDA-LDL (malondialdehyde low-density lipoprotein), ADMA (asymmetric dimethylarginine) and urinary 8-OHdG) were also comparable in the cilnidipine and control groups at these two time points (Table 4).

### 2.4. Effects of Cilnidipine on Power Spectral Analyses of Low-Frequency (LF) and High-Frequency (HF) Activity

With respect to the power spectra, the 24-h, daytime and nighttime low-frequency (LF)/high-frequency (HF) values were similar in the cilnidipine and control groups at baseline (Table 5). However, the 24-h, daytime and nighttime LF/HF increased in the control group, but decreased in the cilnidipine group after the study period, and the differences in these changes were significant between the two groups (Table 5; change in 24-h LF/HF, 0.2 ± 0.4 *versus* −0.1 ± 0.3, *p =* 0.020; change in daytime LF/HF, 0.2 ± 0.4 *versus* −0.1 ± 0.4, *p =* 0.045; change in nighttime LF/HF, 0.1 ± 0.4 *versus* −0.2 ± 0.7, *p =* 0.030).

### 2.5. Effects of Cilnidipine on Parameters of Cardiovascular Function

**Figure 1:**
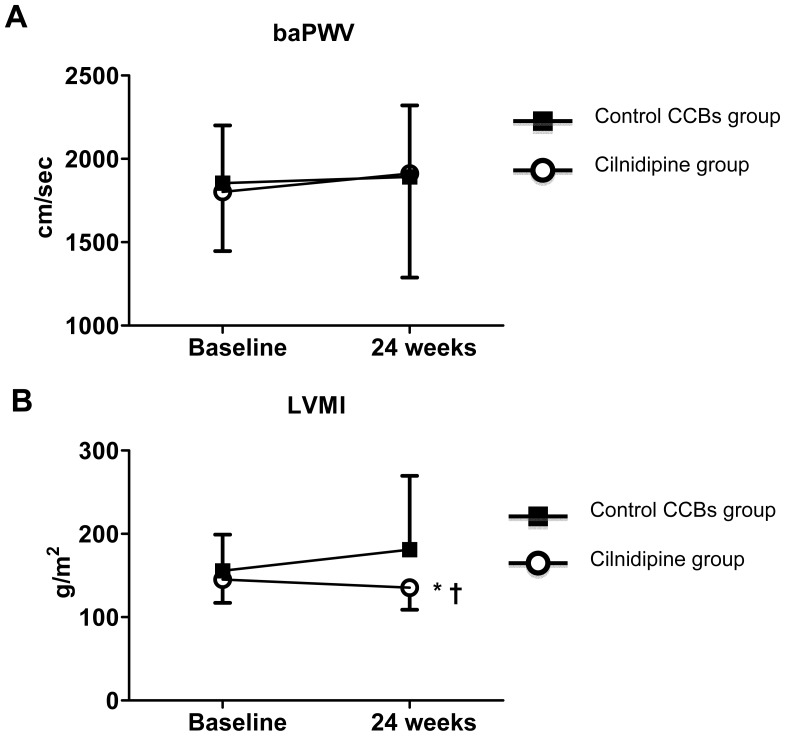
Effects of cilnidipine substitution and the control CCB therapy added to renin-angiotensin system inhibition on brachial-ankle pulse wave velocity (**A**, baPWV) and left ventricular mass index (**B**, LVMI). *p < 0.05 *versus* baseline; ^†^*p <* 0.05 *versus* control group. Values are expressed as the means ± SD.

At baseline, the brachial-ankle pulse wave velocity (baPWV) and LVMI were similar in the cilnidipine and control groups (Figure 1), and the baPWV after the 24-week active treatment period was comparable in the two groups (Figure 1A). On the other hand, the LVMI after 24-week treatment in the cilnidipine group was significantly decreased compared with that in the control group (Figure 1B; LVMI, 135.3 ± 26.4 *versus* 181.2 ± 88.4, *p =* 0.031), with a concomitantly significant difference in the changes in the LVMI between the cilnidipine and control groups (change in LVMI, −12.4 ± 23.7 *versus* 26.2 ± 64.4, *p =* 0.007).

### 2.6. Multivariate Regression Analysis for Assessment of Factors Contributing to Regression of Cardiac Hypertrophy

Multivariate regression analysis of the variables, including age, gender, cilnidipine use, ambulatory BP parameters and an index of power spectral analysis of R-R intervals indicated that there were significant associations between the changes in LVMI and gender and changes in the LVMI and cilnidipine substitution (Table 6). Furthermore, a decrease in ambulatory 24-h LF/HF was suggested as a significant contributing factor to suppression of LVMI in the multivariate regression analysis (Table 6).

## 3. Discussion

The results of the present study show that both the cilnidipine replacement (the cilnidipine group) and continuing the same CCB treatment (the control group) were well-tolerated in hypertensive patients with CKD. With respect to the comparison of the relative BP lowering ability between the two antihypertensive treatment regimens, clinical BP values were similar in the cilnidipine and control CCBs groups. However, with respect to ambulatory BP, the 24-h and daytime systolic BP levels in the cilnidipine group were significantly lower after the 24-week treatment period compared with the control CCBs group, in spite of similar ambulatory BP levels at baseline. Furthermore, the results showed that cilnidipine is superior to the control CCBs in improving left ventricular hypertrophy (LVH).

Previous studies reported that cilnidipine exerted BP lowering effects comparable to other CCBs in hypertensive patients [15–18]. In the present study, although the BP lowering effects on clinical and ambulatory BP were comparable in cilnidipine and the control CCBs, cilnidipine exerted a better inhibitory effect on ambulatory HR. Since cilnidipine is an L/N-type CCB and, thus, may be able to inhibit the reflex activation of sympathetic nerve activity through blockade of N-type calcium channels in the sympathetic nerve endings, this may be one of the advantages of cilnidipine over the other CCBs, such as L-type CCBs, as recently suggested [19].

On the other hand, several previous studies reported that both once-daily L-type CCBs, nifedipine and amlodipine, lowered clinical BP effectively with minimal effects on clinical HR, and there were small differences between nifedipine and amlodipine in the extent to which each activates the sympathetic nervous system with an overall non-significant trend in favor of nifedipine, as estimated by plasma norepinephrine concentrations, power spectral analysis, muscle sympathetic nerve activity and norepinephrine spillover as markers of sympathetic nervous system activation [20,21].

With respect to the property of renal protection afforded by cilnidipine, previous studies showed that cilnidipine had greater antiproteinuric effects than amlodipine, a L-type CCB [18,22], and the results of the Cilnidipine *vs*. Amlodipine Randomized Trial for Evaluation in Renal Disease (CARTER) study showed that cilnidipine was more effective in improving proteinuria than amlodipine in spite of the drugs having comparable clinical BP lowering effects when used as an additional medication for hypertensive CKD patients who had severely increased proteinuria and were undergoing treatment with a RAS inhibitor [17]. In addition, azelnidipine and benidipine, which are dihydropyridine CCBs that block both L-type and T-type calcium channels, are reported to exert superior reducing effects on urinary albumin/protein excretion in hypertensive CKD patients [23,24].

However, although urinary protein excretion after the study period showed a trend of increase in the control group, but a trend of decrease in the cilnidipine group, the difference in these changes was not statistically significant (Table 4). Therefore, the present study did not find a significant reduction in urinary protein excretion in the cilnidipine group (Table 4). The average eGFR of participants in this study was approximately 34–35 (mL/min/1.73 m^2^) and, thus, corresponded to CKD stage G3b, indicating that CKD patients with a more severely impaired renal function had been included in the present study compared to previous studies. This may be a possible reason for the lack of an inhibitory effect of cilnidipine on proteinuria in this study.

On the other hand, the results of the present study showed that the LVMI was significantly improved in the cilnidipine group compared to the control CCBs group after the treatment period (Figure 1). What is the mechanism underlying the efficient inhibitory effect of cilnidipine in hypertensive CKD patients in the present study? The superior inhibitory effect of cilnidipine compared to the control CCBs was accompanied by lower ambulatory LF/HF values after the treatment period (Table 5). Previously, the LF/HF data was suggested to reflect cardiac sympathetic nerve activity, and several studies showed that the LF/HF values were significantly correlated with the LVMI in hypertensive patients [25]. Other studies also showed that the LF/HF values were positively associated with adverse cardiovascular outcomes in other settings, such as post-myocardial infarction, coronary artery disease, congestive heart failure, diabetes and CKD [26–28].

However, while power spectral analysis of R-R intervals (heart rate variability, HRV) has been suggested to reflect sympathovagal imbalance, there is no certainty as to whether changes in HRV are due to changes in sympathetic activity together with changes in vagal component. In the present study, we used the LF/HF values as a possible marker of cardiac sympathetic tone. However, the only study in healthy humans looking at the relationship between HRV and direct measurements of sympathetic activity with cardiac noradrenaline spillover found no significant correlation between 0.1 Hz power expressed in either absolute units or normalized for total power and the rate of noradrenaline spillover from the heart to plasma [29]. This finding was furthermore confirmed by a more recent study in sheep, where direct recordings of cardiac sympathetic nerve activity in normal and heart failure sheep allowed for directly testing whether the difference in cardiac sympathetic nerve activity between the two states is reflected in the LF component of HRV [30]. The results in the sheep study showed that spectral analysis of HRV was not a useful measure of the changes in cardiac sympathetic nerve activity that occur in heart failure [30]. Therefore, since the LF/HF values obtained by power spectral analysis of HRV have not been established as a reliable marker of cardiac sympathetic activity and the results regarding possible inhibitory effects of cilnidipine obtained in this study are principally derived from the ambulatory values of LF/HF, as well as HR, it is important to exercise caution in interpreting the finding to be relevant to an inhibitory property of cilnidipine on cardiac sympathetic nerve activity.

Anti-hypertensive therapy was aimed at achieving the clinical BP goal (BP < 130/80 mmHg), but not the ambulatory BP goal, and thus, the results indeed showed that the mean clinical BP values were similar in the cilnidipine and control groups at baseline and after the 24-week active treatment period (Table 2). However, regarding the ambulatory BP profile, the 24-h and daytime systolic BP levels in the cilnidipine group were significantly lower compared with the control group after the 24-week treatment period (Table 3). In addition, there was an increase in ambulatory HR in the control group, but a decrease in the cilnidipine group (Table 3). Therefore, in spite of the results of the multivariate linear regression analysis in Table 6, the superior inhibitory effect of cilnidipine on LVH compared to the control CCBs may be, at least partly, due to the superior improving property of cilnidipine on ambulatory BP and PR profile compared to the control CCBs.

The limitations of the present study include the relatively small sample size, which may limit the effective determination of significance. The CCBs in the control group included not only the L-type CCBs, but also an L/T-type CCB and an L/T/N-type CCB, thereby making it difficult to strictly compare an L/N-type CCB (cilnidipine) and L-type CCBs, such as amlodipine. In addition, although ARBs and ACEIs have cardio- and renal-protective effects, each drug is reported to have different effects, and there may be different interactions between renin-angiotensin system inhibitors (ARBs, ACEIs and/or DRI) and CCBs. Furthermore, it is important to take the cause of CKD into consideration of the effects of anti-hypertensive treatment. In the present study, there was no significant difference in the cause of CKD between the cilnidipine and control CCBs groups by the analysis using the Chi-square test (Table 1). However, the number of participants in this study was not enough to analyze possible difference in the effects of different CCBs on BP reduction and cardiac and renal function, PRA or norepinephrine, according to the cause of CKD. Further study with a larger sample size is necessary to address these important issues.

## 4. Experimental Section

### 4.1. Study Design

This was a randomized, open-label parallel-group and controlled study (UMIN Clinical Trials Registry: UMIN000001593), and was conducted at the outpatient clinic of the Department of Internal Medicine, Yokohama City University Hospital (Yokohama, Japan). The study consisted of a 2-week run-in period and a 24-week active treatment period. This study was approved by the Ethics Committees of Yokohama City University Hospital, and written informed consent was obtained from every participant.

### 4.2. Study Participants

Inclusion criteria were an age of 20 years or older and the status of being hypertensive CKD patients who were receiving an antihypertensive treatment comprised of renin-angiotensin system inhibitors (ARB, ACEI and/or DRI) and one of the CCBs, other than cilnidipine, at a standard dose for a period of more than 4 weeks. CKD was diagnosed by the presence of albuminuria (*i.e.*, a urinary albumin excretion rate, UACR ≥ 30 mg/g-creatinine) or proteinuria (urinary protein excretion rate, UPCR ≥ 150 mg/g-creatinine or eGFR < 60 mL/min/1.73 m^2^) for a period of more than 3 months. We calculated the eGFR using a revised equation designed for the Japanese population: eGFR (mL/min/1.73 m^2^) = 194 × serum creatinine^−1.094^ × Age^−0.287^ × 0.739 (if female) [31]. Exclusion criteria included severe hypertension (clinical systolic BP ≥ 180 mmHg and/or diastolic BP ≥ 100 mmHg), patients who were on dialysis, women who were nursing or pregnant, hepatic dysfunction or patients who had already been treated with cilnidipine.

### 4.3. Study Treatment

After the run-in period, eligible patients were randomized either to continue the CCBs they were already taking (the control group) or to switch to cilnidipine (the cilnidipine group). In the cilnidipine group, the dose of cilnidipine was titrated as needed to up to 20 mg once daily in the morning during the 24-week active treatment period. In the control group, the doses of the continued CCBs were titrated as needed during the 24-week active treatment period. In both groups, the anti-hypertensive therapy was aimed at achieving, the clinical BP goal (BP < 130/80 mmHg). The doses of renin-angiotensin system inhibitors (ARBs, ACEIs and/or DRI) before the start of trial were not changed during the treatment period.

### 4.4. Clinical BP Measurement and Ambulatory BP Monitoring

The clinical sitting BP was measured at trough (24 ± 2 h post-dose) using a calibrated standard mercury sphygmomanometer and the recommended cuff sizes. Two measurements were taken at 1- to 2-min intervals, and their average was used for calculation purposes. The ambulatory BP and heart rate were monitored every 30 min with a fully automated device (TM-2425, A & D, Tokyo, Japan), essentially as described previously [7,32–34]. The ambulatory BP monitoring was repeated in patients who had >20% values missing, a >30% error rate for the total readings or values missing for more than two consecutive hours. The following readings were omitted, because of technical artifacts: systolic BP > 250 mmHg or <70 mmHg, diastolic BP > 130 mmHg or <30 mmHg, pulse pressure > 160 mmHg or <20 mmHg, systolic differences > 60 mmHg or diastolic differences > 30 mmHg compared with the immediately preceding or subsequent values. The patients were instructed to fill out a diary to record the times of sleeping, rising and other daytime activities. Therefore, the terms “daytime” and “nighttime” in the present study reflect the average period during which the subjects were awake/upright and asleep/supine, respectively. Short-term BP variability, which is based on the coefficients of variation (CV) of the BP values obtained from ambulatory BP monitoring and is suggested to be involved in cardiac injury and hypertrophy [32,35], is defined as the within-subject standard deviation (SD) of all of the systolic and diastolic readings at 30-min intervals, divided by the mean BP during the course of the measurement periods [32–34,36].

### 4.5. Power Spectral Analysis of R-R Intervals for Assessment of Cardiac Sympathetic Nerve Activity

Electrocardiogram (ECG) tracings were obtained with a precordial lead (V5) and ambulatory BP monitoring, and the variation in the R-R interval (RRI) was analyzed as described previously [37]. A total of 512 RRIs were recorded at a resolution of 7.8 ms and were analyzed after fast Fourier transformation using appropriate software (A & D). The power spectral densities of the oscillations were divided into two ranges: an LF component in the range of 0.05–0.15Hz and an HF component in the range of 0.15–0.4 Hz. The LF-to-HF ratio (LF/HF) was calculated as a possible index of sympathovagal balance.

### 4.6. Echocardiography and Brachial-Ankle Pulse Wave Velocity (baPWV)

Echocardiographic studies were performed by trained echocardiographers blinded to the treatment assignment, and the parameters of LV function and hypertrophy, including the LVMI, were measured and calculated as described previously [32–34]. The baPWV values were determined with a PP analyzer (model: BP-203RPEII; Omron Healthcare, Kyoto, Japan), as described previously [34]. Pulse volume waveforms were recorded with sensors placed over the right brachial artery and both tibial arteries. The baPWV values obtained by this method are reported to be significantly correlated with the aortic PWV determined by the catheter method [38].

### 4.7. Laboratory Measurements

Blood and urine sampling was performed between 8 and 10 a.m. after an overnight fast. After the patients had spent 30 min in quiet rest in a recumbent position, blood samples were withdrawn for the determination of the laboratory parameters using routine methods in the Department of Clinical Chemistry, Yokohama City University Hospital. The urinary concentrations of type IV collagen and 8-hydroxydeoxyguanosine (8-OHdG) and the concentration of MDA-LDL and ADMA were determined using an enzyme immunoassay kit (SRL laboratory, Tokyo, Japan) and an ELISA kit (Japan Institute for Control of Aging, Shizuoka, Japan), respectively [7]. The urinary type IV collagen and 8-OHdG concentrations were normalized by the urinary creatinine concentration.

### 4.8. Statistical Analysis

Data were analyzed according to the intention-to-treat principle, and the quantitative data are expressed as the mean ± SD. For the statistical analysis of the difference between baseline and 24 weeks after therapy, analysis of variance was compared by a paired comparison *t*-test. Statistical difference between the groups was assessed by nonparametric analysis using the Mann-Whitney *U* test. Differences between the groups for the categorical variables were analyzed using the Chi-square test. The independent variables that were entered into the multivariate models were the characteristic and ambulatory BP profiles. Analysis was performed with IBM SPSS statistics (version 19, IBM SPSS Statistics, Chicago, IL, USA). A *p*-value <0.05 was considered statistically significant.

## 5. Conclusions

In summary, the results of the present study indicate that cilnidipine replacement improves the ambulatory BP and HR profile by a preferential reduction of the ambulatory BP and HR, and this is suggested to be associated with a significant suppression of LVH in hypertensive CKD patients. Although an inhibitory effect of cilnidipine on cardiac sympathetic nerve activity is an attractive possible property to play a role in this cardiac protective effect, further studies are needed to elucidate the mechanistic basis of the cilnidipine-mediated therapeutic effects on the ambulatory BP and HR profile and cardiorenal function in CKD.

## Figures and Tables

**Table 1:** Patient characteristics. CCB, calcium channel blocker.

Variables	Control CCBs group	Cilnidipine group	*p*

	*n* = 24	*n* = 21	
Age (years)	70.3 ± 11.0	69.5 ± 9.9	NS
Male (%)	54.2	76.2	NS
BMI (kg/m^2^)	24.2 ± 5.0	24.9 ± 4.3	NS
Smoking (%)	45.8	61.9	
Diabetes (%)	37.5	38.1	NS
Dyslipidemia (%)	54.2	42.9	NS
Coronary artery disease (%)	0.0	4.8	NS
Cerebral vascular disease (%)	16.7	19.0	NS

Cause of CKD (*n* (%))			
Nephrosclerosis	14 (58)	15 (71)	NS
Diabetic nephropathy	5 (21)	5 (24)	NS
Chronic glomerulonephritis	3 (13)	1 (5)	NS
Polycystic kidney disease	1 (4)	0 (0)	NS
Chronic pyelonephritis	0 (0)	1 (5)	NS

Medication (%)			
ARBs	91.7	100	NS
ACEIs	20.8	19.0	NS
DRI	4.2	0.0	NS
α blockers	29.2	38.1	NS
β blockers	33.3	14.3	NS
Diuretics			NS
Thiazide diuretics	25.0	23.8	NS
Loop diuretics	16.7	4.8	NS
CNS acting drugs	4.2	0.0	NS

BMI, body mass index; CKD, chronic kidney disease; ARBs, angiotensin receptor blockers; ACEIs, angiotensin-converting enzyme inhibitors; DRI, direct renin inhibitor; CNS, central nervous system; NS, not significant. Data are expressed as the mean ± SD or the percentage.

**Table 2:** Effects of anti-hypertensive therapy on clinical blood pressure (BP).

Clinic BP	Control CCBs group (*n* = 24)			Cilnidipine group (*n* = 21)		

	Baseline	24 weeks	Δ	Baseline	24 weeks	Δ
SBP (mmHg)	142 ± 16	138 ± 16	−3 ± 18	139 ± 16	132 ± 13	−6 ± 14
DBP (mmHg)	81 ± 9	79 ± 7	−1 ± 10	78 ± 13	78 ± 13	0 ± 11
HR (beats/min)	70 ± 10	70 ± 9	−1 ± 9	69 ± 10	69 ± 9	0 ± 11

SBP, systolic BP; DBP, diastolic BP; HR, heart rate. Δ indicates values after 24 weeks minus those at baseline. Data are expressed as the mean ± SD.

**Table 3:** Effects of anti-hypertensive therapy on ambulatory BP profile.

Ambulatory BP	Control CCBs group (*n* = 24)			Cilnidipine group (*n* = 21)		

	Baseline	24 weeks	Δ	Baseline	24 weeks	Δ
24-h						
SBP (mmHg)	133 ± 13	137 ± 15	3 ± 11	131 ± 12	127 ± 9 †	−3 ± 9
DBP (mmHg)	75 ± 8	76 ± 9	1 ± 6	75 ± 8	72 ± 7	−2 ± 6
HR (beats/min)	65 ± 9	68 ± 9	2 ± 5	68 ± 9	64 ± 7 *	−5 ± 9 †
SBPV (%)	12.5 ± 2.7	13.8 ± 2.9	1.3 ± 2.9	14.3 ± 2.7 †	13.5 ± 2.9	−1.3 ± 3.1 †
DBPV (%)	13.7 ± 2.5	14.7 ± 2.8	0.9 ± 2.8	14.4 ± 3.6	15.0 ± 2.9	−0.1 ± 4.4

Daytime						
SBP (mmHg)	136 ± 13	140 ± 15	3 ± 12	134 ± 11	130 ± 10 †	−3 ± 11
DBP (mmHg)	77 ± 9	79 ± 9	2 ± 7	77 ± 8	75 ± 8 *	−2 ± 8
HR (beats/min)	68 ± 15	71 ± 10	3 ± 12	70 ± 7	67 ± 8	−4 ± 6 †
SBPV (%)	12.0 ± 2.6	13.5 ± 3.2	1.3 ± 3.1	13.2 ± 2.9	12.5 ± 2.9	−1.0 ± 3.5
DBPV (%)	13.1 ± 3.0	14.1 ± 3.3	0.7 ± 3.0	13.4 ± 4.0	13.2 ± 3.4	−0.7 ± 4.6

Nighttime						
SBP (mmHg)	126 ± 16	128 ± 18	0 ± 13	122 ± 18	120 ± 11	−2 ± 13
DBP (mmHg)	71 ± 8	71 ± 9	1 ± 8	69 ± 9	67 ± 6	0 ± 7
HR (beats/min)	59 ± 8	62 ± 9	3 ± 6	59 ± 10	58 ± 8	−1 ± 5 †
SBPV (%)	9.4 ± 1.8	9.9 ± 2.2	0.7 ± 2.5	9.7 ± 2.5	11.1 ± 3.4	1.1 ± 4.7
DBPV (%)	10.7 ± 3.3	11.6 ± 4.1	1.2 ± 3.8	11.6 ± 3.2	13.7 ± 3.6	1.3 ± 4.4

SBP, systolic BP; DBP, diastolic BP; HR, heart rate; SBPV, systolic BP variability; DBPV, diastolic BP variability. Δ indicates values after 24 weeks minus those at baseline. Data are expressed as the mean ± SD.

* *p <* 0.05, 24 weeks *versus* baseline.

† *p <* 0.05, cilnidipine group *versus* control group.

**Table 4:** Effects of anti-hypertensive therapy on renal and endocrine function; and inflammation and oxidative stress.

Variables	Control CCBs group (*n* = 24)			Cilnidipine group (*n* = 21)		

	Baseline	24 weeks	Δ	Baseline	24 weeks	Δ
Renal function						
s-Cr (mg/dL)	1.8 ± 1.0	2.0 ± 1.3	0.1 ± 0.5	2.0 ± 1.2	2.1 ± 1.6	0.2 ± 0.6
eGFR (mL/min/1.7 m^2^)	34.9 ± 17.3	34.1 ± 19.3	−0.5 ± 5.4	35.1 ± 17.3	35.0 ± 19.0	−1.1 ± 3.1
UPCR (g/g-Cr)	1.0 ± 1.2	1.1 ± 1.4	0.1 ± 0.9	1.4 ± 2.6	1.1 ± 1.8	−0.2 ± 1.1
Urinary type IV collagen (g/g-Cr)	6.8 ± 3.6	9.2 ± 8.0	2.3 ± 6.1	9.5 ± 11.9	8.4 ± 7.9	−1.1 ± 9.0

Endocrine function						
PRA (ng/mL/h)	2.1 ± 2.4	3.1 ± 4.4	0.9 ± 3.1	2.7 ± 4.3	2.8 ± 3.7	1.0 ± 3.5
PAC (pg/mL)	69.3 ± 44.2	70.7 ± 47.9	−3.9 ± 25.8	66.8 ± 40.6	76.1 ± 55.6	3.6 ± 46.9
Noradrenaline (μg/mL)	393 ± 237	484 ± 275 *	87 ± 168	276 ± 136	364 ± 239	82 ± 272

Inflammation and oxidative stress						
hs-CRP (mg/dL)	0.3 ± 0.6	0.2 ± 0.2	−0.1 ± 0.3	0.1 ± 0.1	0.1 ± 0.1	0.0 ± 0.1
Pentosidine (μg/L)	41.2 ± 25.8	41.0 ± 24.6	−0.8 ± 19.5	41.6 ± 23.0	46.1 ± 27.6	−0.2 ± 18.4
Urinary 8-OHdG (ng/mg-Cr)	4.7 ± 3.5	4.1 ± 2.6	−0.7 ± 3.9	4.1 ± 2.3	4.1 ± 2.3	0.0 ± 3.0
MDA-LDL (u/L)	110.9 ± 51.5	105.0 ± 44.7	−5.9 ± 46.1	93.9 ± 53.7	93.9 ± 42.2	0.0 ± 54.8
ADMA (μmol/L)	0.5 ± 0.1	0.5 ± 0.1	0.0 ± 0.1	0.5 ± 0.1	0.5 ± 0.1	0.0 ± 0.1

s-Cr, serum creatinine; eGFR, estimated glomerular filtration rate; UPCR, urinary protein/creatinine ratio; PRA, plasma renin activity; PAC, plasma aldosterone concentration; hs-CRP, high sensitive C-reactive protein; MDA-LDL, malondialdehyde low-density lipoprotein; ADMA, asymmetric dimethylarginine. Δ indicates values after 24 weeks minus those at baseline. Data are expressed as the mean ± SD.

* *p <* 0.05, 24 weeks *versus* baseline.

**Table 5:** Effects of anti-hypertensive therapy on power spectral analyses of low-frequency (LF) and high-frequency (HF) activity.

Variables	Control CCBs group (*n* = 24)			Cilnidipine group (*n* = 21)		
	Baseline	24 weeks	Δ	Baseline	24 weeks	Δ
24-h LF/HF	1.3 ± 0.4	1.5 ± 0.5 *	0.2 ± 0.4	1.5 ± 0.4	1.4 ± 0.4	−0.1 ± 0.3 †
Daytime LF/HF	1.4 ± 0.4	1.6 ± 0.5 *	0.2 ± 0.4	1.5 ± 0.4	1.4 ± 0.4	−0.1 ± 0.4 †
Nighttime LF/HF	1.3 ± 0.5	1.4 ± 0.6	0.1 ± 0.4	1.5 ± 0.6	1.3 ± 0.7	−0.2 ± 0.7 †

LF/HF, ratio of low-frequency to high-frequency component. Δ indicates values after 24 weeks minus those at baseline. Data are expressed as the mean ± SD.

* *p <* 0.05, 24 weeks *versus* baseline.

† *p <* 0.05, cilnidipine group *versus* control group.

**Table 6:** Multivariate linear regression analysis of changes in LVMI.

Variables	Coefficient	95% interval		*p*

		Lower	Upper	
Age	−0.241	−2.555	0.559	0.200
Gender (Male 1, Female 0)	0.447	9.914	78.272	0.013 *
Treatment group	−0.397	−86.145	−4.755	0.030 *
Changes in 24-h SBP	0.128	−0.095	0.128	0.500
Changes in 24-h DBP	−0.293	−8.390	0.983	0.117
Changes in 24-h HR	0.152	−1.463	3.393	0.422
Changes in 24-h SBPV	−0.302	0.117	−0.302	0.105
Changes in 24-h LF/HF	0.407	7.409	105.041	0.026 *

LVMI, left ventricular mass index; SBP, systolic BP; DBP, diastolic BP; HR, heart rate; SBPV, systolic BP variability; LF/HF, ratio of low-frequency to high-frequency component. Changes indicate values after 24 weeks minus those at baseline.

* *p <* 0.05.
